# Autonomic nervous system dysfunction and sinonasal symptoms

**DOI:** 10.1177/2152656718764233

**Published:** 2018-04-16

**Authors:** Alexander Yao, Janet A. Wilson, Stephen L. Ball

**Affiliations:** From 1 ENT Department, Stepping Hill National Health Service (NHS) Foundation Trust, Stockport, United Kingdom; 2 ENT Department, Newcastle Upon Tyne Hospitals NHS Foundation Trust, Newcastle Upon Tyne, United Kingdom; ^3^Institute of Health and Society, Newcastle University, Newcastle Upon Tyne, United Kingdom No external funding sources reported

## Abstract

**Background:**

The autonomic nervous system (ANS) richly innervates the nose and paranasal sinuses, and has a significant role in lower airway diseases, e.g., asthma. Nonetheless, its contribution to sinonasal symptoms is poorly understood. This review aimed to explore the complex relationship between the ANS and sinonasal symptoms, with reference to systemic diseases and triggers of ANS dysfunction.

**Methods:**

A review of articles published in English was conducted by searching medical literature databases with the key words “autonomic nervous system” and (“sinusitis” or “nose” or “otolaryngology”). All identified abstracts were reviewed, and, from these, relevant published whole articles were selected.

**Results:**

The ANS has a significant role in the pathophysiologic mechanisms that produce sinonasal symptoms. There was limited evidence that describes the relationship of the ANS in sinonasal disease with systemic conditions, e.g. hypertension. There was some evidence to support mechanisms related to physical and psychological stressors in this relationship.

**Conclusion:**

The role of ANS dysfunction in sinonasal disease is highly complex. The ANS sits within a web of multiple factors, including personality and psychological distress, that contribute to sinonasal symptoms. Further research will help to clarify the etiology of ANS dysfunction and its contribution to common systemic conditions.

The autonomic nervous system (ANS) is a phenomenon in health and disease. Its dysfunction transcends a variety of systemic conditions, including cardiovascular and lower airway pathologies.^1,2^ Although the unified airway hypothesis indicates shared pathophysiological processes across both the upper and lower airways,^3–5^ the role of the ANS in nose and sinus symptoms is poorly understood. Historically, the capacity of the nasal vasculature to shrink and engorge in animals was known in the 1850s and was histologically characterized as early as the 1950s.^6^ The important vasomodulatory effects of autonomic nerves in the nose were highlighted by Millonig *et al.*^7^ in 1950 and further supported by evidence from cases of autonomic denervation in patients with nose symptoms. Konno and Togawa^8^ described, in 1979, the successful use of a vidian nerve section to improve symptoms of patients’ allergic rhinitis. However, the transient results and variable benefits for different symptoms indicate a more complex relationship between the nose and the ANS.

It is apparent now that the nose plays an important role in regulating temperature and in humidifying and protecting the passage of air that enters the lower airways *via* the rich autonomic innervation of nasal vasculature and nasal glands.^9–11^ However, the challenges in developing our understanding of the ANS in the nose are compounded by the bewildering variety of tests used to measure ANS function: Valsalva tests, deep breathing tests, isometric handgrip, cold pressor tests, orthostatic tests, tilt-table tests, mental arithmetic, neurotransmitter measurement, sudomotor function tests, microneurography, and heart rate variability (HRV) measurement.^12^

Of these, HRV, as measured through variations between the R waves of the QRS complex (RR interval), has become one of the most accepted and popular tests.^13^ Low frequency (LF) HRV corresponds to both sympathetic activity and parasympathetic activity, whereas high frequency (HF) HRV mainly corresponds to increased parasympathetic activity. Although the LF:HF ratio has been suggested as a measure of sympathovagal balance, this remains highly controversial.^14^ The complexity of the ANS is such that there usually is no single best test and, therefore, tests are more often used in combination rather than in isolation. Recently, increasing evidence that indicates that the characteristic symptoms of sinonasal diseases, including nasal obstruction, rhinorrhea, and facial pressure and/or pain, may be, at least in part, due to disruption of normal ANS function. Furthermore, sinonasal disease has been increasingly linked to chronic systemic disorders, including cardiovascular dysfunction, with the possibility that autonomic dysregulation may underlie these associations.^1^

This review aims to discuss the most recent literature regarding the role of the ANS in the pathophysiology of sinonasal symptoms, including the following: (1) what is the role of the ANS in the causation of sinonasal symptoms; (2) what part does the ANS have in the association of sinonasal symptoms and systemic diseases; (3) what processes drive ANS dysfunction in sinonasal symptoms, in particular, is there a relevant association with physical or psychological stress states? To conclude the clinical implications of these questions will be highlighted. The Medline and Embase (Elsevier, Amsterdam, the Netherlands) data bases searches were conducted by using the Medical Subject Headings key words “autonomic nervous system” and (“sinusitis” or “nose” or “otolaryngology”).

## WHAT IS THE ROLE OF THE ANS IN THE CAUSATION OF SINONASAL SYMPTOMS?

### Nasal Obstruction

**Figure 1.**
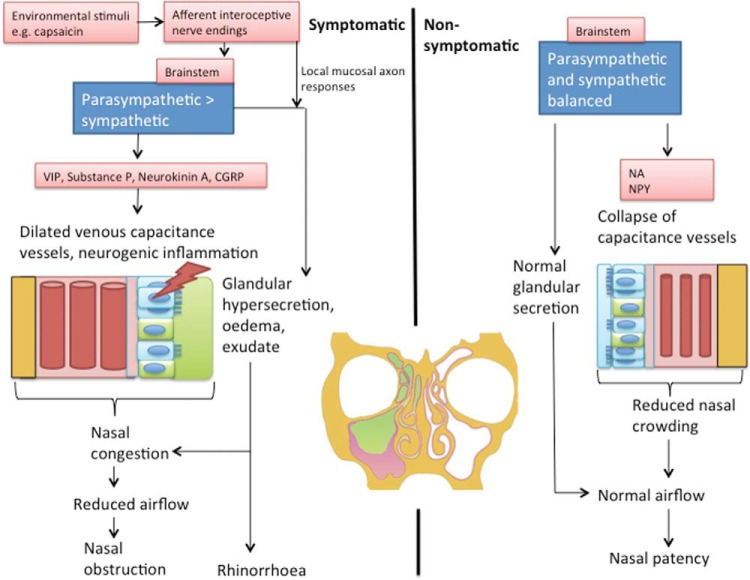
Summary of the pathophysiology of sinonasal symptoms in symptomatic and non-symptomatic individuals. CGRP = calcitonin gene-related peptide; NA = noradrenaline; NPY = neuropeptide Y; TRPV1 = transient receptor potential cation channel subfamily V member 1; VIP = vasoactive intestinal peptide.

Physical nasal obstruction is mediated *via* a variety of mechanisms, including dilatation of the nasal vasculature, mucosal edema, the presence of polyps, and physical deformities, which reduce nasal cavity diameter and thus impede airflow.^11^ Superimposed on this is a degree of sensory modulation that contributes to nasal congestion symptoms, with or without the physical obstruction. A major component of nasal obstruction is the engorgement of the nasal vasculature, which is richly innervated by fibers of the ANS.^10,15^ Sympathetic tone, by causing vasoconstriction and emptying of the venous sinusoids *via* control of arteriovenous anastomoses, is the most important determinant in nasal patency. A reduction in sympathetic tone causes venous sinusoids dilatation and contributes to symptoms of nasal obstruction (Fig. 1).

This sympathetic tone is predominantly mediated by the effect of noradrenaline on the *α*-1 receptors in the venous sinusoids, which act as the capacitance vessels, whereas *α*-2 receptors have a greater role in the control of arteriole constriction and act as the “resistance” blood vessels.^16^ In contrast, *β*-adrenoreceptor stimulation results in vasodilatation and increased blood flow, although the effect of alpha stimulation is more pronounced.^17^ Chemical and surgical attenuation of sympathetic innervation to the nose in both cat models and humans results in nasal obstruction.^18–21^ For example, topically applied *α*-adrenergic antagonists, *e.g.,* phenoxybenzamine, inhibit a-adrenergic–mediated nasalvasoconstriction in cat mucosa. Conversely, stimulation of sympathetic activity in the cervical chain in dogs decreases nasal airway resistance and decreases vessel capacitance.^22^ Humans exposed to upright posture change, exercise, or temperature changes developed increased sympathetic tone, measured with HRV, and improved nasal flow.^23–25^ The response to exercise can be inhibited by injection of anesthetic into the superior cervical ganglion.

Parasympathetic nerve stimulation in the nose has the opposite effect on the venous capacitance vessels and causes their dilatation *via* the action of acetylcholine on M1 and M3 muscarinic receptors as well as releases vasoactive intestinal peptide and nitric oxide, the final mediator of endothelium-derived relaxation factor.^15,17,26^ A small study of 26 patients with vasomotor rhinitis demonstrated HRV, indicated hyperactive parasympathetic activity compared with controls without rhinitis.^27^ Prevention of acetylcholine secretion with botulinum toxin reduces the symptoms of nasal obstruction characteristic of rhinitis.^28^ Both sympathetic and parasympathetic components are suggested to play a role in alternating unilateral nasal obstruction symptoms that behave much like an exaggerated nasal cycle.^17^ It has been demonstrated that attenuation of either sympathetic or parasympathetic innervation to the nose obliterates the nasal cycle phenomenon.

### Nasal Discharge

Nasal discharge is the net result of a combination of plasma extravasation from the dilated vessels, inflammation, cell debris, and secretion from submucosal nasal glands and goblet cells.^29^ Glandular secretion is controlled by the balance of parasympathetic and sympathetic activity in the nose.^30^ Stimulation of parasympathetic activity results in increased nasal secretions rich in lysozyme, immunoglobulin A and glycoproteins.^29^ Electrical stimulation of the feline vidian nerve increases the rate and flow of nasal secretion in a dose-dependent manner.^18^ This effect also occurs when a particular area of the brainstem is stimulated, accompanied by an increase in systemic blood pressure, likely due to simultaneous stimulation of the sympathetic nervous system, which implicates an important role for the brainstem in ANS activity in the nose.

In addition, chemical stimulation with the muscarinic agonist methacholine applied topically was shown to significantly increase nasal secretions in humans.^31^ Patients with chronic rhinorrhea secondary to idiopathic perennial rhinitis demonstrate hypervagal tone on objective autonomic evaluation.^32^ The secretory response is attenuated by chemical or surgical inhibition of cholinergic and vagal stimulation. A Cochrane systematic review, which included seven trials, highlights that ipratropium bromide in patients with common cold have a reduction in rhinorrhea.^33^ Chemical neuromuscular disruption of acetylcholine release with botulinum toxin can also be used to effectively treat refractory rhinitis.^28^ A recent systematic review, which included eight trials, concluded that endoscopic vidian neurectomy is effective at reducing rhinorrhea in patients with intractable non-allergic rhinitis.^34^ These interventions act *via* a common pathway that involves the acetylcholine-stimulated G-protein-coupled M3 muscarinic receptor, which stimulates cyclic guanosine monophosphate and promotes secretion of glandular cells.

Rhinorrhea symptoms similarly represent an imbalance between parasympathetic and sympathetic activity, with a predominance toward parasympathetic hyperactivity. Pupillometric measurement as a measure of autonomic activity indicates a relative parasympathetic hyperactivity with sympathetic hypoactivity in children with allergic rhinitis compared with those without allergic rhinitis.^35^ Similarly, inhibition of sympathetic activity with systemic *α*-antagonists and inverse agonists (*e.g.,* tamsulosin and doxazosin) used for non-nasal conditions have been associated with adverse effects of rhinitis and nasal obstruction.^36,37^

Neuropeptides from autonomic nerves can incite and modulate the inflammatory response and vasodilatation as part of the neurogenic inflammation process. The Cochrane Library defines neurogenic inflammation as the “injurious stimulus of peripheral neurons and resulting release of neuropeptides which affect vascular permeability and help initiate proinflammatory and immune reactions at the site of injury.”^38^ Nasal mucosal inflammation potentially contributes to a variety of symptoms, from obstruction to hyposmia.^39^ The neuromodulatory role of the ANS has been indicated to be an important component in the response toward potential noxious environmental stimuli.^40^

Denervation of autonomic nerves in a rat model suppresses nasal secretion and decreases levels of neuropeptides, including substance P, calcitonin gene-related peptide, vasoactive intestinal peptide, and neuropeptide Y (NP).^41^ A role for these neuropeptides also extends to humans. NPY is a neuropeptide released from adrenergic nerve endings, co-localized with norepinephrine, and acts as a neuromodulator. NPY is regulated, in part, by the inhibitory autoreceptor NPY2 located on presynaptic neurons.^42^ Increased levels of NPY are associated with vasoconstriction, increased nasal patency, and decreased levels of nitric oxide.^43,44^

Substance P also seems to be an important mediator of neurogenic inflammation in conditions such as non-allergic rhinitis.^45,46^ Substance P receptors (neurokinin 1 receptor) are expressed in afferent nerves in submucosal glands and airway mucosa.^47^ A randomized controlled trial that used the substance P-depleting effects of capsaicin demonstrated symptom improvements in patients within non-allergic rhinitis when applied topically for 2 weeks.^48^ Similarly, the topical application of histamine antagonist azelastine is associated with reduced substance P levels and improved vasomotor rhinitis symptom scores.^49^ Recent evidence indicates a role of neuropeptides, such as substance P and bradykinin, in immunomodulation. The neuropeptide calcitonin-related gene peptide is a known potent vasodilator^50^ whose receptors are found in the nose and paranasal sinuses.^51^ Its importance in the autonomic regulation of the vasculature is becoming increasingly recognized.^52^

Histamine released from degranulating nasal mast cells triggers acute allergic rhinitis symptoms,^53^ which can be inhibited by topical antihistamines.^54^ More recent evidence indicates that histamine may also have a role as a neuromodulator of the ANS.^55^ Histamine seems to inhibit the release of norepinephrine from sympathetic nerve endings that innervate the nasal vasculature. Immunohistochemical staining and realtime polymerase chain reaction work carried out on inferior turbinate tissue indicates that histamine acts in this role *via* H_3_ histamine receptors, which causes overall vasodilatation.^55^ The relationship between inflammation and ANS activity is potentially bidirectional because chronic inflammation can upregulate neural hyperresponsiveness and potentiate neurogenic inflammation.^17^

### Sneezing and Reflexes

The importance of the ANS in the immunomodulatory role has long been known.^56^ Similarly, the ANS has an important protective role in airway reflexes that reacts to potentially harmful stimuli. For example, a sneeze-provoking intranasal stimulus mediates the afferent reflex limb *via* H_1_ receptors and the trigeminal nerve.^57^ The efferent response is coordinated by the brainstem *via* multiple central nuclei and pathways. In the first phase of sneezing (nasal-sensitive phase), parasympathetic fibers travel *via* the superficial petrosal nerve and the sphenopalatine ganglion, and cause nasal mucosal secretion. Such responses are bilateral and initiated by parasympathetic fibers that derive from the superior salivary nucleus.^57^ The resultant edema causes trigeminal stimulation and a positive feedback loop, summating neural impulses into the putative sneezing center in the medulla.^58^

When a threshold is reached, the second phase (respiratory phase) is initiated, which results in a systemic stereotyped and coordinated muscular response that involves abdominal, thoracic, pharyngeal, and facial muscles. Anticholinergic agents are able to inhibit the first phase of sneezing. A randomized controlled trial of 411 participants with cold symptoms demonstrated a suppression of the sneeze reflex by intranasal application of the anticholinergic agent ipratropium.^59^ Further evidence from patients with allergic rhinitis supports the role of the ANS in sneezing. In a small study, 10 patients with allergic rhinitis were stimulated with an intranasal allergen, and responses in airflow resistance and HRV were recorded.^60^ After stimulation, nasal airflow resistance increased, accompanied by decreasing LF and the LF:HF ratio, which indicates sympathetic activity withdrawal compared with relatively stable parasympathetic tone.^60^ These findings indicated that sneezing, in addition to rhinorrhea and obstruction, respond to changes in the balance of sympathetic and parasympathetic activity.

The ANS may represent a final common pathway for other nasal reflexes that occur in response to varied provoking stimuli, such as cold, capsaicin, and toxic inhalants.^57^ The attenuation of nasal secretion and vasodilatation responses to stimuli, such as histamine and cold with vidian neurectomy or anticholinergic agents, indicate that these are neurally mediated reflexes.^8,61,62^ Patients with allergic rhinitis demonstrate a heightened symptomatic response to such stimuli compared with healthy subjects without allergy. It is postulated that this phenomenon is caused by lowered sensory nerve thresholds or antidromic stimulation from neuropeptides that are driving neural hyperresponsiveness.^17^ Inhibition of the nasal sensory fibers in the afferent limb of these reflexes with small doses of capsaicin causes desensitization and is associated with decreased levels of the transient receptor potential cation channel subfamily V member 1 (TRPV1) and decreased rhinitis symptoms.^48,63^

### Polyps

There is little evidence for the role of the ANS in polyp disease, although, on histologic examination, the nasal polyp pedicle is richly innervated by sympathetic nerves.^64^

### Facial Pain

The ANS has an important role in cholinergic symptoms associated with facial pain syndromes. Facial pain syndromes, *e.g.,* mid segment facial pain, may pose difficult diagnostic and treatment challenges to the otolaryngologist.^65^ The rhinorrhea seen with trigeminal autonomic cephalgias and migraine are attributable to parasympathetic activation. Blockade of this trigeminal-autonomic reflex *via* surgery or local anesthetic has traditionally been used to successfully treat refractory rhinorrhea and facial pain.^66^ A small randomized controlled trial of 20 patients who underwent sphenopalatine ganglion acupuncture demonstrated significant subjective and objective airway patency improvements compared with sham controls.^43^ More recently, the successful use of oxygen therapy to treat the symptoms of cluster headache has been indicated to be attributable to the effect of oxygen as an anti-inflammatory neuromodulator.^67,68^

## WHAT ROLE MIGHT THE ANS HAVE IN CONNECTING SINONASAL DISEASE TO SYSTEMIC DISEASES?

### Rhinitis and Blood Pressure

The diving reflex phenomenon suggests a neurally mediated connection between nasal stimuli and cardiorespiratory control.^69^ The relationship between rhinitis and blood pressure remains controversial. European-based population studies of 330 men with rhinitis concluded that rhinitis is associated with increased blood pressure in men.^70,71^ However, more recently, a U.S.-based population study of 3912 men and women found no such association.^72^ Interestingly, treatment of allergic rhinitis in patients reduces their systolic blood pressure.^73^ The shared importance of the ANS in nasal symptoms and blood pressure control certainly implicates the possibility that autonomic dysfunction may link both of these conditions, although evidence for causality is currently limited.

### Sinonasal Symptoms and Lower Airways Disease

Sinonasal diseases such as allergic rhinitis and rhinosinusitis have been associated with several lower airway conditions, including asthma and bronchiectasis through the unified airway hypothesis.^74–76^ Cold nasal stimulus and apnea are linked *via* the diving reflex.^69^ However, the role of the ANS in this relationship is not entirely clear. Several physiologic studies over the past 3 decades demonstrate a connection between upper airways inflammation, *e.g.,* in sinusitis or nasal cold stimulus, with lower airway bronchoconstriction through the mechanism of inflammatory seeding or reflex.^77–79^ This response is attenuated by local anesthesia or anticholinergic agents topically applied to the nose.

Our more recent understanding of the unified airway implicates the important role of the ANS in host defense and neuroimmunomodulatory function, *e.g.,* in allergy.^80^ The impact of allergy on neuronal activity is complex and acts on multiple levels, *e.g.,* by increasing sensory nerve excitability, central sensitization, and synaptic efficiency, and by changes to nerve end transmitter release. Potentially harmful stimuli causing C fiber activation in the upper airway (*e.g.,* from nasal or laryngeal mucosa) have been postulated to cause central sensitization of stimuli from the lower airways, which results in increased lower airway reflex responses (such as cough and bronchospasm).^81,82^ The precise mechanisms by which these occur in man remain unclear.

### Sinonasal Symptoms and Gastroesophageal Reflux

Patients with chronic rhinosinusitis (CRS) are more likely to have gastroesophageal reflux disease (GERD) compared with those without CRS.^83^ GERD is also associated with increased symptom burden in patients with CRS.^84^ Moreover, in a cohort of 182 patients who were undergoing functional endoscopic sinus surgery, gastroesophageal reflux was the only condition significantly associated with a poor symptomatic outcome after surgery.^85^ However, there is evidence that an involvement of autonomic dysfunction that links GERD and sinusitis is limited to small studies. In a study of 15 patients, excessive vagal responses were found in those with concomitant asthma and GERD.^86^ Loehrl *et al.*^87^ describe patients with vasomotor rhinitis with or without GERD to have relative adrenergic hypoactivity compared with healthy controls. There was more significant adrenergic hypoactivity in the vasomotor rhinitis group with GERD compared with those without GERD. Although autonomic dysfunction is a feature of GERD and sinonasal disease, there is little explanation offered for the pathophysiology of ANS dysfunction in this association.

### Sinonasal Symptoms and Drug Adverse Effects

Drugs that act on the ANS can have a vasoactive effect on the nose, which causes nasal obstruction and rhinorrhea.^88^ For example, nasal obstruction is a recognized adverse effect of *α*-blockers, *e.g.,* tamsulosin (commonly used in benign prostatic hypertrophy management) and doxazosin (used in hypertension treatment). Other medications causing nasal obstruction adverse effects and include certain psychotropic drugs (*e.g.,* chlorpromazine), peripheral vasodilators (*e.g.,* sildenafil), and hormonal treatments (*e.g.,* estrogen).^88^

## WHAT ARE THE FACTORS AND MECHANISMS THAT DRIVE ANS DYSFUNCTION IN SINONASAL DISEASE?

### Allergy

Host response to external allergen has been postulated to affect autonomic function.^89^ In the sinonasal inflammatory environment, Kubo and Kumazawa^90^ describe a stimulated parasympathetic state, with upregulated muscarinic receptors, and simultaneous adrenergic hypofunction characterized by downregulation of *α*-and *β*-adrenoreceptors.^90^ This phenotype is supported by objective airflow measurement and HRV analysis, which measures ANS function.^60^ Our more recent understanding of molecular neurobiology places an emphasis on the immunomodulatory role of the ANS.

Stimulation with allergenic mediators can also cause long-term changes by increasing neuronal excitability, increasing synaptic efficiency, and modifying gene expression and neuron phenotype.^81^ These changes are consistent with mucosal hypersensitivity described in patients with allergic rhinitis. Noradrenaline released from sympathetic nerve endings act on *α*-and *β*-adrenergic receptors present on macrophages, T cells, B cells, and natural killer (NK) cells.^91^ In the parasympathetic nervous system, vagus afferents are thought to play a role in antigen detection. Parasympathetic efferent activity mediates cholinergic anti-inflammatory properties, which thus completes the “inflammatory reflex.”^92^ Macrophages, for example, express the nicotinic acetylcholine receptor and are highly sensitive to modulation by the cholinergic pathway.

### Stress

Physical or psychogenic stressors may represent an important etiologic factor for ANS dysfunction. Stress and anxiety are associated with allergic rhinitis and CRS.^93–95^ Eccles^96^ noted that psychological stress can cause instability of hypothalamic function and disrupt the autonomic balance in the nasal cycle, migraine, and Meniere’s disease. Psychological stressors, *e.g.,* mental arithmetic or an interview about an upsetting asthma-related incident, were shown to increase parasympathetic activity, which is associated with poor asthma control.^97^ Inhibition of cholinergic function with the anticholinergic agent ipratropium bromide attenuates the increase in airflow resistance after emotionally stressful stimuli.^98^ It has also been suggested that stress *in utero* may program maladaptive neuroimmunomodulatory ANS responses *via* epigenetic effects, predisposing to allergy from an early age.^99,100^

**Figure 2.**
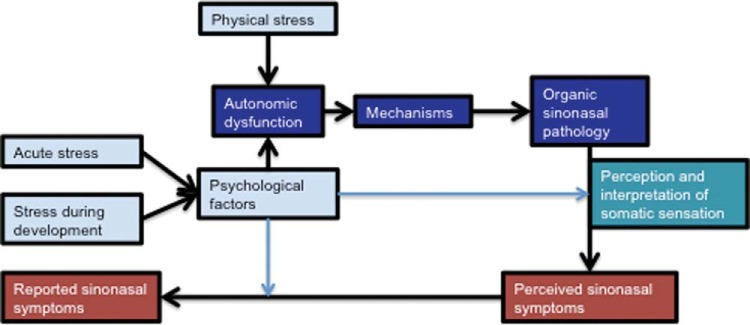
Illustration of the complex relationship between psychological factors, physical factors and sinonasal symptoms.

Physical stressors, such as changes in air temperature, humidity, dust, and noxious chemicals, can induce autonomic-mediated symptoms, such as obstruction and rhinorrhea, in the short term.^101^ However, episodic exposure to temperature extremes may also affect autonomic function over longer periods. A small study, of 26 patients with allergic rhinitis, found that a 6-week course of high-temperature sauna treatment increased sympathetic activity as measured by HRV and increased peak nasal flow.^23^ Overall, it is possible to hypothesize a complex relationship in which psychological stressors and physical stressors drive mechanisms of ANS dysfunction. The resulting pathophysiologic changes as well as personal psychological factors can influence the reporting of sinonasal symptoms (Fig. 2).

## CONCLUSION

The significance of the clinical burden of the ANS on otorhinolaryngologic disorders was highlighted by Hilger^102^ over 60 years ago. However, our understanding of the pathophysiology of ANS dysfunction is limited by mostly level 5 evidence in small studies with a lack of recent updates. Important potential clinical implications urge further research. Characterizing molecular mechanisms in nasal ANS offers potential new targets for intervention within neurogenic inflammation in CRS.^103^ For example, the neuroimmunomodulatory effects of botulinum toxin A showed promise in alleviating symptoms of refractory rhinitis and allergic rhinitis.^28,104^ TRPV receptor antagonists have also emerged as a novel anti-inflammatory class.^105,106^ In addition, a better understanding of the etiology of ANS dysfunction, especially in relation to stress, potentially opens new treatment pathways for patients, *e.g.,* psychological therapy, which includes cognitive behavioral therapy and mindfulness.

The mechanisms from which sinonasal symptoms arise are complex and involve psychological, physical, and biochemical factors. ANS dysfunction is likely to represent just one contributor among many factors that drive sinonasal symptoms. More research is required to (1) establish the direction of causality between ANS dysfunction and sinonasal disease, and (2) determine the etiologic factors that drive ANS dysfunction in the first place.

## ETHICAL APPROVAL

This study was approved by our institutional review board.

## STATEMENT OF HUMAN AND ANIMAL RIGHTS

This article does not contain any studies with human or animal subjects.

## STATEMENT OF INFORMED CONSENT

There are no human subjects in this article and informed consent is not applicable.
